# More Stable Ties or Better Structure? An Examination of the Impact of Co-author Network on Team Knowledge Creation

**DOI:** 10.3389/fpsyg.2017.01484

**Published:** 2017-09-25

**Authors:** Mingze Li, Xiaoli Zhuang, Wenxing Liu, Pengcheng Zhang

**Affiliations:** ^1^School of Management, Wuhan University of Technology Wuhan, China; ^2^School of Economics and Management, China University of Geosciences Wuhan, China; ^3^School of Business Administration, Zhongnan University of Economics and Law Wuhan, China; ^4^School of Management, Huazhong University of Science and Technology Wuhan, China

**Keywords:** tie stability, structural hole, knowledge creation, collaboration, network

## Abstract

This study aims to explore the influence of co-author network on team knowledge creation. Integrating the two traditional perspectives of network relationship and network structure, we examine the direct and interactive effects of tie stability and structural holes on team knowledge creation. Tracking scientific articles published by 111 scholars in the research field of human resource management from the top 8 American universities, we analyze scholars’ scientific co-author networks. The result indicates that tie stability changes the teams’ information processing modes and, when graphed, results in an inverted U-shape relationship between tie stability and team knowledge creation. Moreover, structural holes in co-author network are proved to be harmful to team knowledge sharing and diffusion, thereby impeding team knowledge creation. Also, tie stability and structural hole interactively influence team knowledge creation. When the number of structural hole is low in the co-author network, the graphical representation of the relationship between tie stability and team knowledge creation tends to be a more distinct U-shape.

## Introduction

As knowledge is important to the development of society and organizations, there is a burgeoning interest on how to create more knowledge in scientific research (Lambiotte and Panzarasa, 2009). Traditionally, scholars have focused on the role of individual personality or talents on knowledge creation (e.g., Bowler and Morus, 2010). However, recent knowledge management researchers are shifting their attentions from the individual factors to team factors (Wuchty et al., 2007). Given knowledge creation is becoming more and more complex, researchers build teams in order to meet their knowledge creation goals. This shift poses a challenge for researchers: how can teams manage the process of knowledge creation successfully?

The majority of research adopts the paradigm of “input-process-output” model to explore the antecedents and process of team knowledge creation. Following this model, researchers suggest that team diversity such as educational background, gender, age diversity (Smith et al., 2005), leadership behavior (Nonaka et al., 2006) and organizational policies (Argote et al., 2003) are critical antecedents of team knowledge creation. They also identify team learning (Stacey, 2001), team members’ motivations (Sosa, 2011) and feedback (Akbar, 2003) as key processes that stimulate team knowledge creation. However, prior research mainly focuses on the effects of teams’ cognition or behaviors among team members on team knowledge creation. This approach fails to capture the influence of team members’ interactions on team knowledge creation. Team knowledge creation refers to a continuous, self-transcending process during which team members obtain, absorb and integrate valuable external knowledge through their interaction with others (Nonaka et al., 2000). This process emphasizes team members’ interactions (Schumpeter, 1934; Polanyi and Sen, 1967). Thus, some researchers introduce social networks theory to investigate team knowledge creation.

Social network theory offers theoretical lens to analyze the influence of embedded relationship on individual or team’s behavior. Generally, previous studies explore network effects manly from two different perspectives (Moran, 2005). The first perspective focuses on the direct tie effects such as tie strength (i.e., the mean frequency interactions among actors) on organizational outcomes (e.g., Labianca and Brass, 2006). The second perspective, from a macro level, posits that the network structure such as network density (i.e., ratio of extant edges to potential edges) plays the most important role on shaping individual or team’s behavior (e.g., Galaskiewicz and Burt, 1991; Burkhardt, 1994). These two streams have pushed social network study forward tremendously. However, due to the lack of comparative and comprehensive study on the two perspectives, we know little about the exact role that network structure and directive tie states play in the team knowledge creation process. Importantly, we do not know whether these two aspects have interactive effects on team knowledge creation. Hence, this study attempts to address this knowledge gap by choosing specific variables from these two different perspectives and comparing the direct effects of these variables while examining the interactive influence of the two network perspectives on team knowledge creation.

Existing studies of direct ties mainly focus on the effect of interactive frequency among actors, i.e., tie strength, on knowledge creation. For example, McFadyen et al. (2009) find that average tie strength is one of the critical factors that influencing knowledge creation. However, the majority of these studies have overlooked the time aspect of the ties. This is problematic because the same interaction between two actors may occur in 1 day, it may also happen in 1 month or even 1 year. If researchers only focus on frequency, there is no way for us to know if the ties among actors are stable. Further, we will not clear about whether tie stability (i.e., keeping a certain relationship for a long time) will benefits team knowledge creation. Therefore, we will first examine the relationship between tie stability and team knowledge creation.

Considering the studies focus on network structures, researchers mainly emphasize two critical variables, i.e., network density and centrality. For example, network density, defined as the proportion of potential ties in a network that are actually present (Ahuja et al., 2012), has been identified as impeding factor of knowledge creation (McFadyen et al., 2009), and centrality, defined as the extent to which a network revolves around a single node, has been proven to have positive effects on knowledge creation (Matusik and Heeley, 2005). However, structural hole, referring to the acts that serve as mediators between two or more closely connected groups, has been considered as an very important attribute of network structure (Burt, 1992), few studies have examined the relationship between structural hole and team knowledge creation. In addition, both attributes of tie and structures may interactively influence team knowledge creation. To our knowledge, few studies have examined the interactive effect of tie stability and structural hole on knowledge creation. Therefore, the second aim of this study is to examine the direct effect of structural hole as well as the interactive effect of tie stability and structural hole on team knowledge creation.

The present study contributes to the knowledge creation literature in two aspects. Firstly, despite some links existing between ego network and individual knowledge creation (e.g., Smith et al., 2005), we know surprisingly little about how new knowledge is created in teams (McFadyen and Cannella, 2004; McFadyen et al., 2009). This study sheds light on this aspect by identifying the influences of co-author network on team knowledge creation. Secondly, we intend to integrate the two different perspectives, i.e., network structure (structural holes) and network tie attribution (tie stability), to examine the direct and interactive effects of the two on team knowledge creation. Prior studies either examine the effect of tie attribution such as tie strength on knowledge creation (e.g., Levin and Cross, 2004), or identify the network structure, such as density and centrality on knowledge creation (e.g., Gilsing et al., 2008). There is no theoretical and empirical evidence of how these two aspects of network interact simultaneously. This study adds value on the influence of co-author network on team knowledge creation.

## Theoretical Background

Knowledge creation is a continuous, self-transcending process during which individuals obtain, absorb and integrate valuable external knowledge through their interaction with others (Nonaka et al., 2000). It is affected by individuals’ current knowledge system and external knowledge processing environment. This process, to some extent, is an information processing process. Although individual and collective processes of knowledge creation are similar, the only difference between them is that the individual process emphasizes the integration of knowledge in one’s mind; whereas the collective process emphasizes the interaction among team members Lavie and Drori (2012). Information is the input to teams and new knowledge output via the interactions among team members. Collective information processing theory involves a prerequisite assumption that task-relevant information is acquired and shared among team members (De Dreu et al., 2008). In other words, team members comprehend and process the new information they have acquired from each other or the external world and form a new collective understanding of the real. Hence, if all members are regarded as an information processing agent, team knowledge creation can be further recognized as a collective information process. We define team knowledge creation as a process of collaborative group performance, during which team members collectively amplify the knowledge created by some individuals and crystallize it as part of the knowledge system of the team (Nonaka et al., 1996; Mitchell et al., 2009).

Team members’ interactions allow information transfer, process and development into common cognitive at the team level. Klein and Kozlowski (2000) elaborate two ideal modes of the emerging of collective knowledge in team information processing. The first one is composition. Composition emphasizing the assumptions of isomorphism and treats, regards team cognition as a convergence of similar cognitive properties at the individual level. It describes the generating process of new knowledge from the lower-level to the higher-level and the consistency of individual knowledge and systematic cognition. The second mode is compilation. Based on assumptions of discontinuity, compilation describes the combination and restructuring of differentiated knowledge during information processing by emphasizing essential functions of differentiated knowledge. Therefore, we suggest that these two information processing processes supplement each other in team knowledge creation. In particular, composition process emphasizes the integration of homogeneous knowledge, thereby forming the optimal solution, compilation process, which underlines the role of heterogeneous knowledge, ensures that knowledge could be extended.

## Hypotheses

### Effect of Tie Stability on Team Knowledge Creation

We define tie stability as the proportions of team members who maintain a long time cooperative relationship with others (Huggins, 2010). The higher the tie stability is, the larger the proportions of members who have maintained a long time cooperative relationship with other team members. Tie stability emphasizes the time aspect of the tie rather than the frequency aspect. Some scholars find that stable relationships may enhance the transfer of tacit knowledge and thus be beneficial for knowledge creation (Ebadi and Utterback, 1984; Moran, 2005), while others argue that changes in cooperative relations motivate a team to transform its conventional thinking, thereby helping maintain knowledge heterogeneity and promoting team members to generate novel ideas (Choi and Thompson, 2005). Also, according to similar theory, if team members interact with each other too long or too frequently, the information or knowledge they possess will step toward a similar trend (Lewis et al., 2007).

We believe tie stability is like a double-edged sword in that it will change teams’ information processing modes. According to the collective information processing theory, composition process emphasizes the identical facet of knowledge, believing homogeneity is the basis for the combination of heterogeneous knowledge. By contrast, compilation process emphasizes the heterogeneity aspect of knowledge, regarding the variety of knowledge as the impetus for knowledge development and deepening (Klein and Kozlowski, 2000). We assume that team knowledge creation could not be realized only with one of these two processes. Alternatively, only when the two processes reach a balanced proportion can collective knowledge creation be effectively promoted. The reason lies in that the homogeneous aspects of knowledge provide convenience for knowledge combination (Pinjani and Palvia, 2013), whereas, the heterogeneous aspects of knowledge provide possibility for knowledge expansion (Swan et al., 1999).

As mentioned previously, tie stability represents the proportions of members who have maintained an enduring cooperative relationship with other team members. If a team’s tie stability is high, indicating that the team members’ interaction with each other is frequent, this condition is beneficial for knowledge combination within the team. However, too much interaction among team members may lead to a similar tendency of their knowledge and thinking (Huggins, 2010), which may impede their knowledge expansion and further harm team knowledge creation (Lewis et al., 2007). In other words, tie stability may determine the information process mode in the team, which in turn influences the team knowledge creation. When tie stability is low, a context for developing heterogeneous knowledge, will promote the information processing mode of compilation. By contrast, when tie stability is high, a context for developing homogeneous knowledge (Huggins, 2010), will trigger the information processing mode of composition. Therefore, if tie stability is moderate, compilation and composition may reach a balance, this context will greatly benefit to team knowledge creation. We propose the following hypothesis:

Hypothesis 1: There is a U-shaped relationship between tie stability and team knowledge creation. Specifically, moderate tie stability benefits team knowledge creation, whereas lower and higher tie stability result in poor performance in team knowledge creation.

### Structural Holes and Team Knowledge Creation

The concept of the structural hole was established by Burt (1992). It describes social networks where two or more individuals build indirect connections by connecting to a third party but no direct relationships exists between them. Prior research suggests that structural hole brings many advantages and conveniences to individuals who occupy the position of structural holes. For instance, Burt (2004) points out that individuals occupying structural holes can embrace more opportunities of gaining information or resource from others as they bridge two or more individuals. Frankort (2008) notes that structural hole elevates individuals’ performance and creativity as it reduces information redundancy and provides people more opportunities to access heterogeneous information. Nevertheless, with regards to team knowledge creation, structural holes might do more harm than good.

As mentioned previously, team knowledge creation can be regarded as a collective information processing process. This process emphasizes information and knowledge sharing. Structural hole focuses on the relationship of team members reach out to each other by the third party rather than by direct connection. This indirect connection undoubtedly results in difficulty in information flows between them. If a team contains many structural holes, the proportion of team members’ non-direct communication will increase. As a few members within the structural holes largely control the internal information of a team, knowledge and information sharing will become difficult. Furthermore, information transferred through the third party may result in some distortion, thereby hindering internal team information flows. Obstfeld (2002) suggested that the increase in structural holes inevitably affects team creativity as the structural holes indulge team members’ opportunistic behaviors, which obstructions for the transmission of information. Therefore, a team with more structural holes tends to have more difficulties in information sharing, giving rise to disadvantages for team knowledge creation. Following this analysis, we propose the second hypothesis:

Hypothesis 2: Structural hole in co-author network is negatively related to performance quality of team knowledge creation.

### Interactive Effect of Tie Stability and Structural Holes

As mentioned above, the degree of co-author network tie stability determines the proportion between homogeneous and heterogeneous elements of information transfer among team members. The number of structural holes influences the fluency and efficiency of knowledge exchange. According to information processing theory, information processing, basing on information sharing and exchanging among team members (De Dreu et al., 2008), is a critical factor for team knowledge creation. Specifically, when a team enjoys high efficiency in information transfer and sharing, the speed in integrating its homogeneous and heterogeneous knowledge or information can be accelerated, which leads to improvement of the efficiency in team knowledge creation. By contrast, when the sharing of team information is hindered, the homogeneous and heterogeneous information is not exchanged effectively, and team knowledge creation is impeded.

As mentioned previously, structural hole influences the efficiency of team knowledge creation by disturbing information sharing and exchange process within the team. If a team’s co-author network includes too many structural holes, information transfer will be difficult among team members (Ahuja, 2000), and will impede team knowledge creation. Although tie stability may increase the possibility of information exchange among team members, this beneficial effect may be offset by the negative impact of high structural hole. Also, as a team’s tie stability increases, team members increasingly interact within the team, leading team members’ information and thinking to a similar trend and thus hindering team knowledge creation (Huggins, 2010). However, studies indicate that structural hole can increase heterogeneous information, because it increases possibility of accessing information from different parties (Burt, 2004). Hence the negative effect caused by high stability may also be offset by high structural holes. Therefore, in teams with high structural hole, the U-shaped relationship between tie stability and team knowledge creation would be weakened and trend to be more linear.

The increase of co-author network’s tie stability in an appropriate extent will benefit information transfer and exchange within the team, thereby promoting team knowledge creation. However, if a team’s co-author network possesses the low structural hole, information transfer efficiency will benefit (Balkundi et al., 2007). Hence, the positive relationship between tie stability and team knowledge creation in the appropriate extent will be strengthened in teams with low structural hole. In addition, if a team’s tie stability increases to an excessive extent, team members’ increased interaction will lead to homogeny of team members’ information, and thus hinder team knowledge creation (Huggins, 2010). A low structural hole context which also benefits to information transfer within the team may also strengthen the negative effect caused by high tie stability. Based on the arguments above, we propose the third hypothesis.

Hypothesis 3: The inverted U-shape relationship between tie stability and team knowledge creation is moderated by the number of structural holes. Specifically, when there are less structural holes, the inverted U-shape relationship between the two would be amplified; when there are more structural holes, this relationship would be significantly weakened, and trending to be a more linear relationship.

## Method

### Sample and Procedure

First, we selected eight top academic institutions in the United States based on the widely recognized rankings by experts in human resources management. Then, we accessed school websites of these eight academic institutions to obtain the names and resumes of scholars in human resources management. Through this process, we gathered 191 qualified scholars. By collecting their published papers from 2005 to 2009 on the Institute for Scientific Information (ISI) database and tracking their coauthors, we captured every scholar’s research co-author network. As the data on impact factors of journals were relatively complete from 2005 to 2009, we designated these 5 years as our research period. Since scholars may use different surnames and abbreviated forms while publishing during their academic career, to achieve a complete data set, we searched all different probable surnames and abbreviated forms within the given period in ISI database. Through the process of screening and data, we identified 111 scholars in these eight academic institutions. Starting from these scholars we identified and tracked co-authors to develop our co-author networks for research. The sample includes 862 scholars and 591 published academic papers. In addition to this, we also recorded information such as authors’ names, gender, paper titles, journal titles, publishing year, impact factors for journals, citation frequency, years after obtaining Ph.Ds.

We also would like to note that the data is objective and valid from ISI website. Also, the research has been performed in accordance with the recommendations of the Science and Technology Research Office of Huazhong University of Science and Technology. There were no unethical behaviors in the research process, and we were exempt from further ethics board approval since our study did not involve human clinical trials or animal experiments.

### Measures

#### Tie Stability

Tie stability refers to the degree of stable co-author relations in networks. Prior researchers have proposed a similar variable concept. For example, McFadyen et al. (2009) has applied “number of long-term coauthors” to measure collaboration relationships that lasted for 6 years or more. It is a way to measure the number of members who maintain stable co-author relations with others. However, we deem that time of collaboration is also an important embodiment of tie stability. Given that completing two papers in a top journal must be a long time commitment (usually more than about 4 years), we assumed that if two scholars have published two or more papers together, they maintain a stable relationship. We calculated the proportion of stable relationships to represent the tie stability of team (i.e., the number of team members who have published two papers with the same co-author divided by the number of team members). Based on this calculation, the minimum value of the ratio is “0,” denoting that no stable ties exist among coauthors; while the maximum is “1,” meaning that all of the coauthors in network are maintaining stable relationships.

#### Structural Holes

We employed research methods proposed by Burt (1992) to calculate the number of structural holes in co-author networks. Using matrix data of the co-author network, we adopted Ucinet 6 social network analysis software to calculate structural holes index of each team network.

#### Team Knowledge Creation

We adapted quality and quantity as two criteria for the evaluation of team knowledge creation. We used the journal’s impact factors of each publication to assess quality. For quantity, we used the total number of papers published. Then we calculate the impact factors for all of the articles published to evaluate team knowledge creation. The journal impact factors considered were the values reported for the publication year of each study. McFadyen et al. (2009) also used impact factor of journals to access knowledge creation.

#### Control Variables

To control differences in scholars’ genders (male = “0,” female = “1”) and knowledge, we included gender, years after gaining Ph.Ds., the ratios of first authored and last authored publications as control variables. Besides, as tie strength (the interactive frequency between two actors; Granovetter, 1983) is a variable which is similar to tie stability, we control the tie strength of network members to differentiate the influence of tie strength and tie stability on team knowledge creation.

## Results

### Descriptive Statistics

**Table 1** presents the mean, standard deviation and correlation coefficients for each variable. It shows that among the scholars, the average number of years after gaining a Ph.D. was 17.53 years, and 71% of those scholars were male. Besides, tie strength was positively related to tie stability (*r* = 0.45, *p* < 0.01), and tie stability had positive correlation with team knowledge creation (*r* = 0.27, *p* < 0.01), structural holes displayed a strong negative correlation with team knowledge creation (*r* = 0.27 = -0.60, *p* < 0.01). These result preliminarily supported our hypothesis 2.

**Table 1:** Mean, standard deviation, and correlations.

	Mean	*SD*	1	2	3	4	5	6	7
(1) Gender	0.71	0.46							
(2) Years after obtaining Ph.D.	17.53	10.8	0.01						
(3) Proportion of first authored papers	0.42	0.34	-0.01	0.01					
(4) Proportion of last authored papers	0.27	0.30	-0.01	0.07	-0.51^∗∗^				
(5) Tie strength	0.61	0.28	0.12^∗^	0.03	0.27^∗∗^	-0.08			
(6) Tie stability	0.20	0.26	0.13	0.03	-0.03	0.00	0.45^∗∗^		
(7) Structural holes	0.55	0.30	-0.02	0.16	0.04	0.15	0.10	-0.01	
(8) Team knowledge creation	12.61	13.74	0.16	-0.15	-0.05	-0.00	0.27^∗∗^	0.27^∗∗^	-0.60^∗∗^


*N = 111, ^∗^p < 0.05 (two-tailed test), ^∗∗^p < 0.01 (two-tailed test)*.

### Regression Analysis

**Table 1** shows that the mean of “years after gaining Ph.D.” is 17.53, and the mean of “team knowledge creation” was 12.61, which were far larger than the average mean of other control variables and independent variables. In order to reduce the bias of estimation, we first used logarithm to address “years after gaining Ph.D.” to diminish difference in mean, and then ran negative binomial regressions to analyze the data. Before the analysis, we standardized all independent variables in case of multicollinearity. In the following step, we entered all control variables and added tie stability and its quadratic term into the model to examine hypothesis 1. Then we added structural hole to examine hypothesis 2. To test hypotheses, we further added interaction terms of structural holes and tie stability into the model. Detailed results of the negative binomial regression are reported in **Table 2**.

**Table 2:** Results of regression analysis.

Variables		Team knowledge creation				
		M1	M2	M3	M4	M5
Control variables	Constant	3.30^∗∗^	3.11^∗∗^	3.01^∗∗^	2.26^∗∗^	2.12^∗∗^
	Gender	0.23	0.21	0.17	0.16	0.16
	Years after gaining Ph.D.	-0.27^∗^	-0.26^∗^	-0.17^∗^	-0.11	-0.10
	Proportion of first authored papers	-0.63	-0.34	-0.11	-0.02	0.19
	Proportion of last authored papers	-0.30	-0.07	0.26	0.33	0.40
	Tie strength	0.48^∗∗^	0.31^∗^	0.26^∗∗^	0.45^∗∗^	0.34^∗∗^
Predictive variables	Tie stability		0.31^∗^	0.81^∗∗^		0.25^∗∗^
	Tie stability^2^ (H1)			-0.43^∗∗^		-0.12
	Structural holes (H2)				-0.81^∗∗^	-0.83^∗∗^
	Tie stability^∗^structural holes					-0.25^∗∗^
	Tie stability^2∗^structural holes (H3)					0.13^∗^
	Pseudo *R*^2^	0.03	0.04	0.10	0.18	0.20
	ΔPseudo *R*^2^		0.01	0.06		0.02
	LR chi^2^	27.57^∗∗^	32.77^∗∗^	77.83^∗∗^	138.76^∗∗^	155.99^∗∗^


*N = 111, ^∗^p < 0.05 (two-tailed test), ^∗∗^p < 0.01 (two-tailed test)*.

Hypothesis 1 predicts an inverted U-shape relationship between tie stability and team knowledge creation. Statistically, if the regression coefficient of tie stability squared is negative, significant and the model goodness of fit is better than the controlled model, this hypothesis will be supported. As shown in **Table 2**, the coefficient of tie stability quadratic term was negative and significant (β = -0.43, *P* < 0.01, *Model 3*). Meanwhile, relative to Models 1 and 2, adding quadratic term of tie stability accounts for 0.07 and 0.06 increase of ΔPseudo R squared statistic, respectively (ΔPseudo *R*^2^= 0.06; LR chi^2^= 77.83, *P* < 0.01; *model 3*) indicating a better goodness of fit. Thus, Hypothesis 1 was supported. Hypothesis 2 predicts that the number of structural holes is negatively related to team knowledge creation. Statistically, if the regression coefficient of structural hole is negative and significant and the model goodness of fit is better than the controlled model, this hypothesis will be supported. As shown in **Table 2**, the coefficient of structural holes was negative and significant (β = -0.81, *P* < 0.01, *Model 4*). Compared to control model, the goodness of fit of the model was increased significantly (Pseudo *R*^2^ = 0.18; LR chi^2^ = 138.76, *P* < 0.01, *Model 4*), thus supporting hypothesis 2.

**FIGURE 1:**
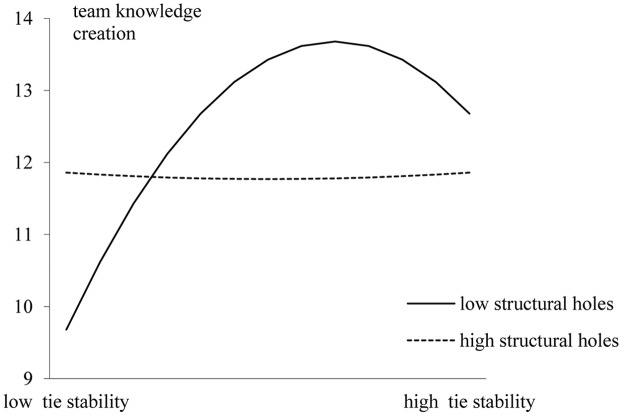
Interaction Effect of Structural Holes and Tie Stability (±2*SD*).

Hypothesis 3 predicts that the interaction between tie stability and structural holes has impact on team knowledge creation. Statistically, if the regression coefficient of tie stability squared × structural hole is significant and the model goodness of fit is better than the controlled model. In addition, the interactive graph pattern trends consist with the proposition, then, this hypothesis will be supported. As shown in **Table 2**, Model 5 shows that the coefficient of the interaction term of tie stability squared × structural was significant (β = 0.13, *p* < 0.05; *Model 5*). When the interaction term were entered, Pseudo *R*^2^ indexes increased 50% relative to Model 3 and 11.1% (Pseudo *R*^2^ = 0.20; LR chi^2^ = 155.99, *P* < 0.01, *Model 5*) relative to Model 4. In order the present the whole trends of the interactive pattern, we used two standard deviations above and below the mean of tie stability and one deviation above and below structural hole as criteria to plot the interaction diagram (Cohen et al., 2013). As shown in **Figure 1**, when the structural hole was low, the inverted U-shape relationship between tie stability and team knowledge creation became more distinctive; In contrast, when structural hole was high, the relationship between the two became flatter, and displayed a more linear shape. These results provide support for Hypothesis 3.

## Discussion

### Theoretical Implications

As a crucial cognitive resource in organizational management, knowledge creation occupies a pivotal position in the knowledge management field. Scholars appeal further exploration to this issue so as to reveal its internal mechanism and important factors. To compensate for the limitations of prior research in psychological and cognitive perspectives, we applied the social network perspective and combined ideas of collective information processing theory to examine the interactive effect of research co-author networks and structure on team knowledge performance. Our study has extended previous research in several aspects:

First, the present research focuses on the impact of tie stability on team knowledge creation in co-author networks. Extensive research at the micro level concentrates on the influence of tie strength or relation object on creative thinking (Baer, 2010). Tie strength reflects the tightness of direct interactions among team members, emphasizing the communication frequency among cohorts (Granovetter, 1983). Our study illustrates tie stability among group members and the general flow of coauthor-network and its percentage from tie stability perspective. It contributes to previous research by applying a new scope to analyze the effect of tie on team knowledge creation and extends our understanding of this issue.

Second, previous studies have generally examined the effects of network centrality, network density and number of sub-groups on team performance (e.g., Brass et al., 2004). In contrast, this study selects indexes of structural holes as research variable, which enriches our understanding of network structural effects on team knowledge creation. Though sparse studies have explored structural holes, they tend to focused on individual level and drew positive conclusions as researchers believe individuals occupying structural holes have the advantages of accessing to more and different information and resources (Soda et al., 2004). Approaching from a team level scope, our study provides evidence that the number of structural holes has negative effect on team knowledge creation, revealing the dark side of structural holes.

Finally, by combining the perspectives of co-author networks tie state and structure pattern, we seek to explore the interactive effect of the two on team knowledge creation. Although few previous studies have examined the two perspectives, respectively (e.g., Smith et al., 2005), studies approach from the comprehensive view are rare. We find evidence that tie stability and structural hole would interactively influence team knowledge creation by intervening the information processing process within the team. The results indicate that the effect of tie state, such as stability, on team knowledge creation might be weakened or strengthened by network structural pattern, such as structure hole. Prior studies either explore the effect on team knowledge creation from the perspective of tie state or from the perspective of network structural. These studies have identified that both tie state and structure pattern have significant impact on team knowledge creation. However, prior studies overlook that this two perspectives may have interrelationship. The present study examining the interactive effects of the two different perspectives provides new understanding of the relationship between network and team knowledge creation.

### Managerial Implication

Knowledge is created during individuals’ interaction with others rather than generated in isolation (Phelps et al., 2012). Only through comparing his or her own idea with others’ can individual improve their understanding of specific issues. From this perspective, knowledge creation is team work. Therefore, the relational schema of team members’ co-author networks must affect team knowledge creation. The results of this study also suggest some managerial implications for organization practice. Firstly, team members need to maintain both stable and flowing relations with others properly. Stable tie is a foundation for team members to form convergent and integrated knowledge; while tie state provides team with heterogeneous knowledge resources and information. Organizations need to balance the homogeneous and heterogeneous knowledge formed as a result of tie stability to help team members synthesize information.

Further, the network structure among team members determines the efficiency of knowledge transfer and sharing which are the foundations of knowledge integration, influencing team knowledge creation Reagans and McEvily (2003). For business organizations, advantages in policies need to be given a full play to shape the collaborative networks among team members and thus to develop the structural benefits of cooperative networks. For example, research teams can properly adjust and shape network coauthor relation to reduce the occurrence of structural holes and increase the density of co-author networks tie to increase the speed and efficiency of knowledge and information sharing.

Finally, by combining the perspectives of co-author networks tie state and structure pattern, we seek to explore the interactive effect of the two on team knowledge creation. A few previous studied have examined the two perspectives, respectively (e.g., Smith et al., 2005), while studies approached from the comprehensive view are rare (Phelps et al., 2012). We find evidence that tie and structure have interactive influencing on team knowledge creation. Both Tie stability and structural hole can influence the efficiency of team knowledge and information sharing and transfer, and hence have impact on team knowledge creation. In other words, co-author network direct tie and structure pattern have interactively influence on team knowledge creation.

## Limitations And Future Research Directions

Although the present study brings significant insights into this research topic, it also has several limitations. Firstly, as our research examined the coauthor state among team members based on a given period, it is unable to reveal the dynamic state of co-author networks. Future research can be done from a comparative study of coauthor state in different periods to reveal how changes in networks affect the performance of team knowledge creation. Next, the use of coauthored publications to track network membership in our study reflects the members’ interactions to some extent, yet fails to reflect tacit communication completely. Scholars may conduct their future studies with the combination of interview and questionnaire to provide deeper insights. Moreover, the research indexes selected in our study is relatively limited. In the context of enough samples and with the ability to overcome the difficulty in obtaining resources, future studies can make a more comprehensive investigation on knowledge creation performance by adopting more network indexes and by combining non-network index factors drawn from previous research. Furthermore, the inferences made about managerial implications are a bit of a stretch, as this study cannot really tell us much about creative process caused by tie stability. The implications should focus more tightly on what this might cause about academic publishing and networks of authors who publish together frequently. For example, the mediators of the relationship between tie stability and team knowledge creation are worth to be investigated in future research. Last, it should be acknowledged that 71% of the sample was Male. This seems extraordinary, particularly in the domain of human resource management. Thus, randomness of this sample must be reconsidered in the next study.

## Author Contributions

ML contributed in finishing the first draft. XZ revised this paper. PZ and WL contributed to this research by giving suggestions and involved in data collection.

## Conflict of Interest Statement

The authors declare that the research was conducted in the absence of any commercial or financial relationships that could be construed as a potential conflict of interest.
